# Optimal detection of hypothyroidism in early stage laryngeal cancer treated with radiotherapy

**DOI:** 10.1186/s40463-015-0085-3

**Published:** 2015-09-11

**Authors:** Graeme B. Mulholland, Han Zhang, Nhu-Tram A. Nguyen, Nicholas Tkacyzk, Hadi Seikaly, Daniel O’Connell, Vincent L. Biron, Jeffrey R. Harris

**Affiliations:** Division of Otolaryngology-Head and Neck Surgery, University of Alberta Hospital, 1E4.29 WMC, 8440 – 112 Street, Edmonton, AB T6G 2B7 Canada; Division of Radiation Oncology, McMaster University, Hamilton, Canada; Northern Ontario School of Medicine, Sudbury, Canada

**Keywords:** Hypothyroidism, Early stage laryngeal squamous cell carcinoma, Radiation therapy

## Abstract

**Background:**

Hypothyroidism following radiation therapy (RT) for treatment of Head and Neck Cancer (HNC) is a common occurrence. Rates of hypothyroidism following RT for Early Stage Laryngeal Squamous Cell Carcinoma (ES-LSCC) are among the highest. Although routine screening for hypothyroidism is recommended; its optimal schedule has not yet been established. We aim to determine the prevalence and optimal timing of testing for hypothyroidism in ES-LSCC treated with RT.

**Method:**

We conducted a population-based cohort study. Data was extracted from a prospective provincial head and neck cancer database. Demographic, survival data, and pre- and post-treatment thyroid stimulating hormone (TSH) levels were obtained for patients diagnosed with ES-LSCC from 2008–2012. Inclusion criteria consisted of patients diagnosed clinically with ES-LSCC (T1 or 2, N0, M0) treated with curative intent. Patients were excluded if there was a history of hypothyroidism before the treatment or any previous history of head and neck cancers.

**Results:**

Ninety-five patients were included in this study. Mean age was 66.1 years (range: 44.0–88.0 years) and 82.3 % of patients were male. Glottis was the most common subsite at 77.9 % and the average follow-up was 40 months (Range: 12–56 months). Five-year overall survival generated using the Kaplan-Meier method was 79 %. Incidence of hypothyroidism after RT was found to be 46.9 %. The greatest frequency of developing hypothyroidism was at 12 months.

**Conclusions:**

We found a high prevalence of hypothyroidism for ES-LSCC treated with RT, with the highest rate at 12 months. Consequently, we recommend possible routine screening for hypothyroidism using TSH level starting at 12 months. To our knowledge, this is the first study to suggest the optimal timing for the detection of hypothyroidism.

## Introduction

Head and neck cancer (HNC) encompasses 3 % of total malignancies in North America, with a large proportion presenting as laryngeal squamous cell carcinoma (LSCC) [1]. One thousand and fifty cases were diagnosed alone in Canada in 2014 [2]. Treatment for early stage LSCC (ES-LSCC) has traditionally utilized single modality regimes consisting of either radiation therapy (RT) or surgical resection [3, 4]. Due to increasing advances in radiation planning over the past decade using computerized tomography based image planning, many cancer treatment centers including ours have adopted RT as the preferred method of treatment for ES-LSCC [5].

Despite improvements in RT therapy, its effects on the thyroid gland remains significant, as it is located in very close proximity to the target of treatment [6, 7]. Hypothyroidism as the result of radiation induced fibrosis and compromise of thyroid vascularity is still a common unnoticed complication after treatment of LSCC with a frequency of 14–36 % [8]. The most common signs and symptoms of hypothyroidism present as dry skin, cold sensitivity, fatigue, muscle cramps, voice changes, and constipation [9]. Left untreated hypothyroidism is associated with increased total low-density lipoprotein cholesterol and cardiac morbidity including mortality and atherosclerotic events [10]. As a result of these risks, the NCCN guidelines recognise the risk of developing hypothyroidism in association with treatments for LSCC and recommend screening every 6 to 12 months following treatment [11]. However, to our knowledge there has been no conclusive evidence looking at the optimal time in screening for hypothyroidism for ES-LSCC treated with RT. Therefore, we sought to investigate the ideal time of testing for hypothyroidism in patients with ES-LSCC treated with RT defined as the point in time where the test is associated with the highest frequency. We also evaluated the incidence of hypothyroidism in this patient population within Alberta, Canada.

## Method

Ethics approval was granted by the University of Alberta’s Health Research Ethics Board (HREB) and the Alberta Cancer Board.

### Patients

Inclusion criteria were defined as: residents of Alberta greater than 18 years of age, with biopsy-proven early stage (T1 or T2, N0) LSCC (based on the 7^th^ Edition of the AJCC TNM Staging Manual) [12]. All treated using primary RT with curative intent in Alberta.

Exclusion criteria were defined as: patients with previous HNC with or without treatment, a diagnosis of hypothyroidism prior to radiation treatment or incomplete data sets from chart review.

### Data collection

All patients diagnosed with ES-LSCC meeting inclusion criteria from January 1, 2008 to December 31, 2012 were included in the study. Demographic, survival and clinicopathologic data was obtained initially through the Alberta Cancer Registry (ACR) by a data analyst. The ACR is a population-based registry established in 1942 that records and maintains data of all new cancer cases, their treatments, and resulting deaths in the province of Alberta in a longitudinal and prospective fashion [13]. A review of outpatient, inpatient, and cancer clinic records was then performed for quality assurance and to extract relevant patient, tumour, lab values (pre-treatment albumin and pre and post-treatment thyroid stimulating hormone (TSH) levels), treatment, follow-up, as well as survival data Charlson Comorbidity Index (CCI) scores, which were not included in the ACR database, were calculated using relevant comorbidities taken from chart review [14]. Date of diagnosis was defined as the date of pathologically confirmed ES-LSCC.

### Staging

Staging of the tumours was clinical and according to the seventh edition of the American Joint Committee on Cancer (AJCC) TNM staging manual [12].

### Treatment

All patients underwent radiation therapy (RT) for curative intent. Patients receiving RT or CRT for distant metastases or palliation were not included. Intensity-modulated or 3-D conformal RT were utilized with dosing between 60.75 and 70 Gy, using 2 Gy per fraction, depending on the T-status [11].

### Outcomes

The primary outcome was set as the optimal time of testing for the elevation of TSH after treatment of ES-LSCC defined as the point in time where the testing is the most sensitive. The time interval to the initial elevation of TSH after treatment of ES-LSCC with RT was calculated for every patient. This was defined as the time from completion of RT until the first elevated TSH. The secondary outcome was the incidence of hypothyroidism after treatment of ES-LSCC with RT.

### Hypothyroidism

All included patients had pre-treatment TSH values. Patients were then screened for elevated TSH levels at 3–6 month intervals starting at 3 months until 30 months. Hypothyroidism was defined as an elevated TSH value based on the reference value given for each type of TSH test (TSH only (0.20–4.00 mU/L) or Progressive TSH (0.30–4.00 mU/L).

### Follow-up

All patients were followed at regional cancer treatment centers at regular intervals following treatment. Our cut off time point was February 1^st^, 2015. Patients who were suspected to have disease recurrence underwent a metastatic workup including appropriate imaging, endoscopy and biopsy as per standard of care.

### Statistical analysis

Baseline characteristics were compared using standard modes of comparison. Continuous data was analyzed using analysis of variance (ANOVA). Categorical data was compared using the chi-squared test. Univariate analysis was performed to determine the prevalence of hypothyroidism and the optimal timing of the elevation of TSH. Analyses were performed using SPSS Statistics 20.0 (SPSS Inc, Chicago, IL).

## Results

One hundred and sixty two patients were diagnosed with ES-LSCC in Alberta from 2008 to 2013. Of these, 67 patients were excluded: 11 for insufficient data points, 8 patients were treated with primary surgery and 48 patients had an elevated TSH prior to the start of RT treatment. Exclusion criteria were then applied to inclusion criteria, leading to a final data set of 95 patients for analysis.

**Fig. 1:**
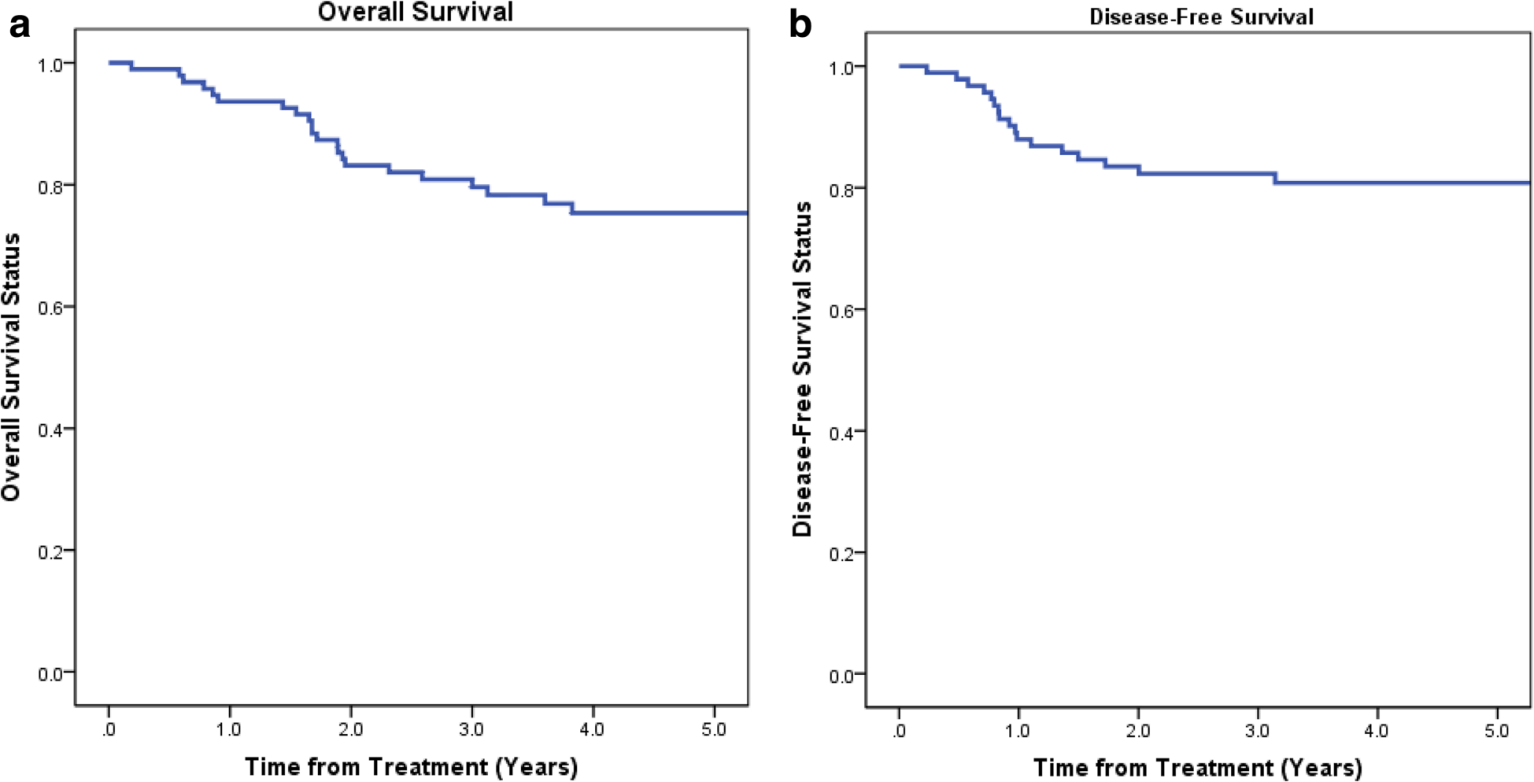
**a** Overall Survival, **b** Disease-Free Survival rates for patients with stage I or II laryngeal cancer

Figure 1 represents overall and disease-free survival for our ES-LSCC data set. Five-year overall and disease-free survivals were 79 and 81 % respectively.

Table 1 contains patient demographic and tumour subsites information. The average age was 66.1 years (range: 41.0–88.0 years) with a strong male predominance (82.3 %). Glottis was the most common tumor subsite (77.9 %), followed by subglottis (14.7 %). A minority of cases were represented by supraglottis and transglottis subsites at 4.2 and 3.2 % respectively.

**Table 1:** Patient characteristics

Variable	N
n	95
Mean Age (range), years	66.1 (41.0–88.0)
Gender, no. (%)	
Male	79 (82.3)
Female	16 (16.7)
Mean CCI (range)	2.8 (0.0–13.0)
Tumour Subsite, no. (%)	
Glottis	74 (77.9)
Subglottis	14 (14.7)
Supraglottis	4 (4.2)
Transglottic	3 (3.2)
T-Stage, no. (%)	
1	59 (61.4)
2	37 (38.6)

*CCI* Charlson comorbidity index

**Fig. 2:**
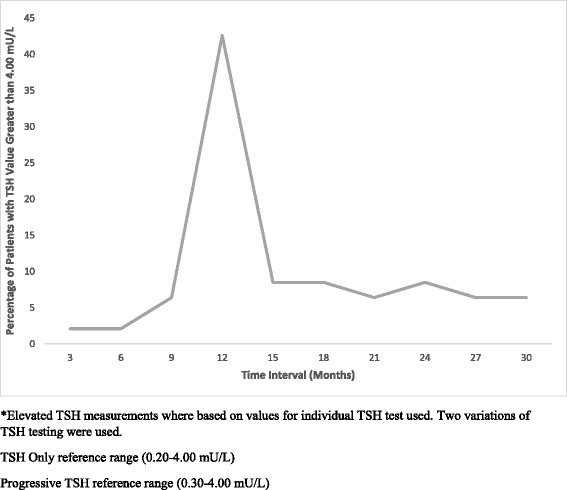
Patients with TSH Greater than 4.00 mU/L by Time of Presentation

Tables 2 shows TSH specific data. Forty five (46.9 %) of patients had elevated TSH during the period of follow up. In addition, 27 patients were found to have a TSH greater than 10.00 mU/L. Figure 2 shows the breakdown in terms of time to elevated TSH. The majority of patients (42 %) had elevated TSH at the 12-month interval.

**Table 2:** Hypothyroidism variables

Variable	N
Elevated TSH, no. (%)	45 (46.9)
Mean Peak TSH (range), mIU/mL	16.9 (4.0–55.9)
Number of Patients TSH > 10 mU/L	27

*TSH* Thyroid stimulating hormone

**Fig. 3:**
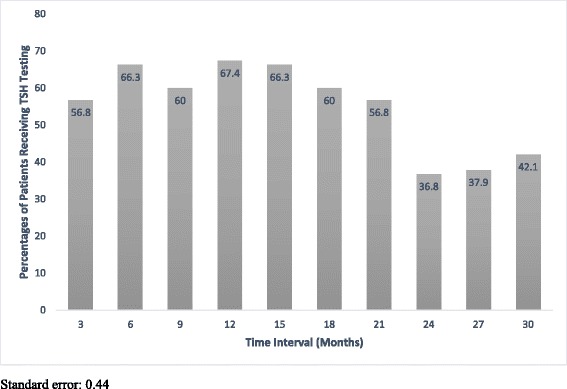
Percentage of Patients Receiving TSH Testing at Given Time Intervals

Seventy six of 95 (80.0 %) and 79 of 95 patients (83.2 %) received TSH testing within the first 15 and 18 months following initiation of RT. Of the 19 patients not receiving TSH testing during the first 15 months following treatment 6 patients presented with elevated TSH values upon first TSH testing. Figure 3 illustrates the distribution and frequency of TSH screening. Testing started at 3 month and was continued through 30 months after completion of RT treatments. The majority of TSH testing took place within the first 21 months. For TSH levels tested at 3, 6, 9, 12, 15, 18 and 21 months the frequency of patients tested was greater than 50 % for all intervals (56.8, 66.3, 60.0, 67.4, 66.3, 60.0 and 56.8 %) respectively. From 24 to 30 months between 36.8 and 42.1 % of patients received TSH screening. A standard error calculation was performed for this data with a value of 0.44. The single greatest screening interval was at 12 months, where 67.4 % of patients had TSH testing. The lowest screening interval was at 24 months with 36.8 % of patients tested.

## Discussion

It is well established that treatment of HNC using RT, chemotherapy or surgery increases the risk of developing hypothyroidism. Based on the literature, compared to other treatments modalities for ES-LSCC, RT is associated with the greatest risk, 48 % at 5 years and 67 % at 8 years following treatment [15]. Its pathophysiology is not completely understood, but most likely involves damage to thyroid vasculature. Additional theories specific to RT involve direct micro and macrovascular damage, fibrosis of the thyroid capsule which may limit compensatory thyroid enlargement and even the formation of induced antithyroglobulin antibodies [7]. The risk of developing hypothyroidism becomes greater the closer the anatomical relationship between the treated tissue and thyroid tissue [16]. Despite this high incidence, no study has looked at hypothyroidism in patients who receive single modality therapy with RT for ES-LSCC. As such, there are currently no standardised post treatment hypothyroidism screening recommendations specifically for this patient population.

The literature has yielded multiple studies investigating the effects of RT in the treatment of HNC and LSCC within a heterogeneous patient population. Two of the most relevant studies are discussed. A systematic review published in 2011 by Boomsma et al. [17] looking at the incidence of hypothyroidism in all presentations of HNC treated with RT cited rates of subclinical hypothyroidism from 23 to 53 % at median follow up times of 2.4 to 6.1 years post RT. They found that hypothyroidism developed at a median interval of 1.4 to 1.8 years after treatment. The paper suggests that the development of subclinical hypothyroidism found at median follow up times should act as an indicator for the duration of TSH monitoring following treatment. However, no follow up regime or optimal time to initiate screening was suggested. The study by Kumar et al. [18] is the best example of a population similar to the one we examined. They looked at all curative treatment modalities including RT, surgery as well as chemoradiotherapy (CRT) for ES-LSCC and found rates of subclinical hypothyroidism at 24 % and symptomatic hypothyroidism at 6 %. Unfortunately, they were unable to provide information related to timing of onset of hypothyroidism post RT. Information in relation to the length of follow up was also insufficient as a result of limited access to patient TSH results and follow up clinical information. In comparison, we found 46.9 % of our study group developed elevated TSH following treatment with RT. This is most commonly observed at the 12 month mark with 89.4 % of patients whom developed and elevated TSH at or after this time point.

Screening for hypothyroidism with TSH is reliant on a normal functioning hypothalamic–pituitary–thyroid axis. The use of TSH is cheap, reliable and sensitive to fluctuations in thyroxine (T4) levels [9]. Our study used TSH as a marker for hypothyroidism, identifying cases of thyroid gland dysfunction. Subclinical hypothyroidism (mild thyroid gland failure) is defined as persistently elevated TSH with normal thyroxine (T4) levels over a period of at least 3 months. Overt hypothyroidism relies on a persistently elevated TSH with low T4 levels [19]. Prolonged elevation of TSH levels correlate with significant long term sequelae. Increased total low-density lipoprotein cholesterol and cardiac morbidity including mortality and atherosclerotic events are among the most severe [10]. Therefore, thyroid hormone replacement therapy is recommended in all cases where TSH is > than 10 mIU/L [9]. Given the strong correlation between TSH values and significant long term sequelae, at our institution, the use of TSH alone suffices in determining need for treatment as well as for use as a maker of thyroid dysfunction.

Utilizing TSH ensured sufficient data for collection and ease of comparison from one institution to another. Reference ranges for elevated TSH varied depending on institution or type of test (progressive TSH or TSH only). All elevated TSH tests were categorized as hypothyroidism as measured by reference ranges for each test. We obtained mean peak TSH values of 16.9 mIU/L (4.0–55.9 mIU/L) in our population indicating a need for thyroxine replacement to avoid long term sequelae. Status and treatment of clinical versus sublinical hypothyroidism was beyond the scope of our study. Within our patient cohort, access to TSH information was more reliable than T3/T4 which was not routinely done as part of the hypothyroidism work up. Additionally, a protocol for treating subclinical versus clinical hypothyroidism is still not well defined. From the literature, a study looking at presentations of subclinical versus biochemical hypothyroidism in 2 randomized controlled trials by Murthy et al. [20] examined 122 patients affected by head and neck cancers treated with 3D conformational RT. They measured TSH and T4 levels every 3 to 6 months following completdraw ion of treatment. Patients with elevated TSH alone were deemed subclinical hypothyroidism and patients with elevated TSH and low T4 levels were classified as biochemical hypothyroidism. Hypothyroidism presented in 55 % of patients at a median follows up of 44 months, of these 39.3 % subclinical and 15.7 % biochemical. As described by the author, the decision to treat patient was not based on T4, rather treatment initiation was based on presentation with symptoms of hypothyroidism or prolonged elevation of TSH. This is in keeping with the literature. A TSH level greater or equal to 10 mIU/L for a period longer than 3 months is deemed significant enough to initiate thyroid hormone replacement therapy in order to obviate long term consequences. Therefore for our own population of ES-LSCC treated with RT, TSH was utilized alone as the cut-off point to avoid long term sequelae.

Based on our results, the optimal time to initiating screening for hypothyroidism is at 12 months. We observed a significant peak at this time point represented by 20 cases (42.6 %). The earliest elevated TSH was noted at 3 month in a minority of cases (2.1 %). It should be noted the majority of patients presented with elevation of TSH at or after 12 months (87.3 %). The incidence of elevated TSH in the Albertan ES-LSCC population was 45 patents (46.9 %). We found evidence that thyroid function worsens with time, the mean peak TSH was observed at 26.6 months with an average TSH value of 16.9 mIU/L. The delay in presentation in peak TSH is likely explained by a combination of factors. Thyroid stimulating hormone values greater than 4 mIU/L and less than 10 mIU/L are indeterminate in terms of initiating treatment [9]. The onset of hypothyroidism following RT is insidious and increases significantly with time following treatment [15]. Likely, the development of TSH level greater than 10mIU/L appeared more frequently at a greater duration of time following RT. Also, time for treatment optimization and patient compliance with treatment (routinely elevated TSH levels do not produce symptomatology) may contribute to the delay in peak TSH values. Given that our study indicates a peak TSH a later time point than the first observed elevation of TSH and that the risk of developing hypothyroidism increases with time following treatment. This may reinforce the need for lifelong TSH screening. Not only to pick up hypothyroidism initially but to titrate treatments appropriately.

Limitations of the study are acknowledged. The prospectively collected, retrospectively reviewed population-based design of the study did not allow for controls. No set protocol for TSH screening following RT is in place throughout Alberta. Although patients generally receive screening in an every 6–12 month fashion the number of screening events are at the discretion of the family physician, radiation or surgical oncologist. The majority of screening took place between 3 and 21 months. At each of these time intervals greater than 50 % of patients received screening (Fig. 3), a good indication that although no explicit screening regime is implemented, the majority of patients received routine investigation for hypothyroidism. Screening tapered after 21 months with between 36.8 and 42.1 % patients being screened between 24 and 30 months. Additionally, we were only able to consistently access TSH results, consequently without T4 results we were unable to definitively classify patients into categories of subclinical versus overt hypothyroidism. Even without T4 information available TSH information was able to indicate the need for treatment in our ES-LSCC population. Our study also lacked fields of RT therapy for individual patients, while this may have led to confounding of data, all patients were staged at T1/2 and N0 with standardized curative RT protocol as per the Division of Radiation Oncology at the Cross Cancer Institute, University of Alberta. Given these limitations a multi-institutional, prospectively planned study utilizing a set hypothyroidism screening regime—including TSH and T4 monitoring at set time intervals—would be useful to determine whether hypothyroid screening protocols affect patient outcomes.

## Conclusion

Hypothyroidism in patients with ES-LSCC treated with curative RT is common. This population-based study suggests that optimal timing for screening for hypothyroidism using TSH is at 12 months’ time. Future prospective studies to develop screening protocols for hypothyroidism in patients with ES-LSCC treated with curative RT should be undertaken.
